# Purification, crystallization and characterization of the *Pseudomonas* outer membrane protein FapF, a functional amyloid transporter

**DOI:** 10.1107/S2053230X16017921

**Published:** 2016-11-30

**Authors:** Sarah L. Rouse, Wlliam J. Hawthorne, Sebastian Lambert, Marc L. Morgan, Stephen A. Hare, Stephen Matthews

**Affiliations:** a Life Sciences, Imperial College London, South Kensington, London SW7 2AZ, England; b Biological Sciences, National University of Singapore, 14 Science Drive 4, Singapore 117543, Singapore

**Keywords:** amyloid transporter, bacterial outer membrane, FapF, *Pseudomonas*, C8E4

## Abstract

Bacteria often produce extracellular amyloid fibres *via* a multi-component secretion system. Aggregation-prone, unstructured subunits cross the periplasm and are secreted through the outer membrane, after which they self-assemble. Here, significant progress is presented towards solving the high-resolution crystal structure of the novel amyloid transporter FapF from *Pseudomonas*, which facilitates the secretion of the amyloid-forming polypeptide FapC across the bacterial outer membrane. This represents the first step towards obtaining structural insight into the products of the *Pseudomonas* *fap* operon. Initial attempts at crystallizing full-length and N-terminally truncated constructs by refolding techniques were not successful; however, after preparing FapF^106–430^ from the membrane fraction, reproducible crystals were obtained using the sitting-drop method of vapour diffusion. Diffraction data have been processed to 2.5 Å resolution. These crystals belonged to the monoclinic space group *C*121, with unit-cell parameters *a* = 143.4, *b* = 124.6, *c* = 80.4 Å, α = γ = 90, β = 96.32° and three monomers in the asymmetric unit. It was found that the switch to complete detergent exchange into C8E4 was crucial for forming well diffracting crystals, and it is suggested that this combined with limited proteolysis is a potentially useful protocol for membrane β-barrel protein crystallography. The three-dimensional structure of FapF will provide invaluable information on the mechanistic differences of biogenesis between the curli and Fap functional amyloid systems.

## Abstract 2

FapF from *Pseudomonas* facilitates the secretion of the amyloid-forming polypeptide FapC across the bacterial outer membrane. A protocol for forming reproducible well diffracting crystals is described in which the switch to tetraethylene glycol monooctyl ether detergent during purification proved to be critical and represents a handy tip for β-barrel protein crystallography.

## 1. Introduction   

Amyloids, fibrillar proteinaceous aggregates with a cross-β-strand structure, are commonly associated with human disease states such as Alzheimer’s and Parkinson’s. However, certain bacteria have evolved to exploit such fibres to their advantage by secreting monomeric subunits in a highly controlled manner and allowing their self-assembly as part of a biofilm matrix. This matrix assists them in attachment to their host (animal or surface), persistence in infection and antibiotic resistance. Understanding the molecular basis for controlled amyloid formation is of great interest, not only for new strategies to counter bacterial infection, but also to inspire the design of therapeutic approaches to pathogenic amyloidogenesis in humans. The model systems that have been best characterized to date are curli from *Escherichia coli* and functional amyloid protein from *Pseudomonas* (Fap). These two systems form morphologically similar fibres despite being genetically distinct (Dueholm *et al.*, 2010 ▸).

The curli system has been extensively studied, with structures available for all but one of the encoded proteins [CsgA (Tian *et al.*, 2015 ▸), CsgC (Taylor *et al.*, 2011 ▸), CsgE (Shu *et al.*, 2016 ▸) and CsgG (Cao *et al.*, 2014 ▸; Goyal *et al.*, 2013 ▸, 2014 ▸)]. In contrast, no structural information exists for the more recently discovered Fap system. Both of these systems require the polypeptide amyloid subunit to be transported across the complex bacterial outer membrane (OM). In curli, the membrane component CsgG was found to be a nonameric β-barrel in which each monomer contributes four β-strands to a central 36-residue OM channel. The integral membrane component from the *fap* operon, FapF, is similarly predicted to be a β-barrel membrane protein; however, bioinformatics analyses suggest that its structure is likely to be more akin to the fatty-acid transporter β-barrels [*e.g.* FadL (van den Berg *et al.*, 2004 ▸) and TodX (Hearn *et al.*, 2008 ▸)]. This suggests that the two membrane components are distinct, raising the intriguing prospect that the mechanism of amyloid secretion is completely independent. Structural predictions using *PSIPRED* (Bryson *et al.*, 2005 ▸) indicate that FapF comprises an ∼30-residue helical region at the N-terminus, followed by an ∼60-residue disordered region and then an integral membrane β-barrel component.

**Figure 1:**
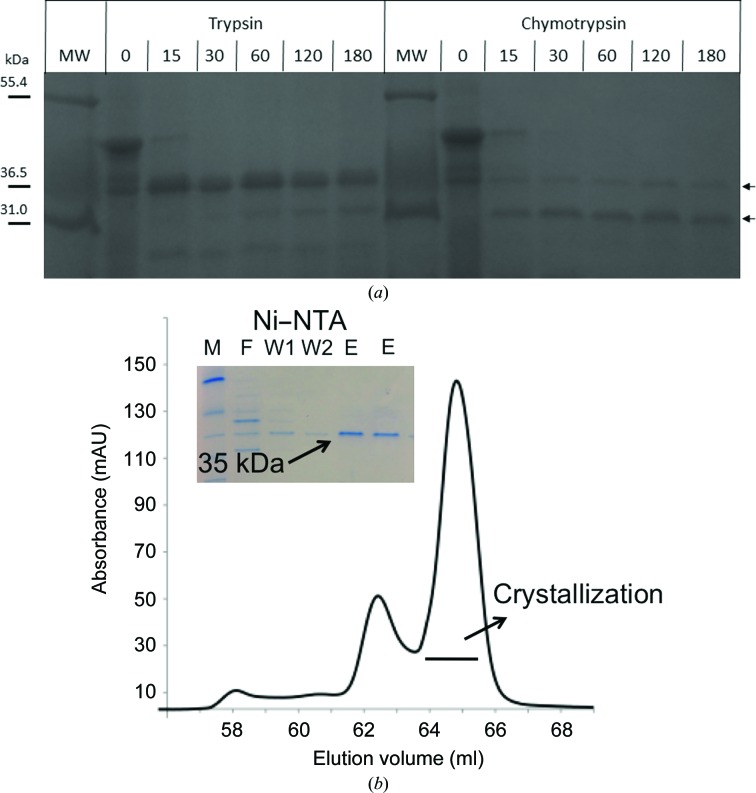
(*a*) Limited proteolysis of FapF. SDS–PAGE gel showing samples of FapF digested over a range of time periods with trypsin and chymotrypsin, with samples taken periodically over the course of 180 min as indicated (labelled in minutes). The protein is processed to produce stable fragments of approximately 35 or 31 kDa (indicated with arrows). Lane MW contains molecular-weight marker (labelled in kDa). (*b*) Purification of FapF. Superdex 200 (GE Healthcare) gel-filtration profile of FapF^106–430^ in 0.5% C8E4. The main peak at 65 ml corresponds to FapF^106–430^. Inset, SDS–PAGE of fractions from the FapF^106–430^ nickel purification: M, marker; F, flowthrough; W1, first wash; W2, second wash; E, elution peaks 1 and 2.

We set out to solve the structure of the FapF outer membrane protein. As our initial efforts to crystallize full-length FapF proved challenging, N-terminally truncated constructs were designed based on molecular-weight information from limited proteolysis (Fig. 1 ▸). These were expressed and purified from the outer membrane fraction of *E. coli* and used for crystallization trials. A complete detergent exchange into tetraethylene glycol monooctyl ether (C8E4) during chromatographic purification was essential for forming well diffracting crystals and suggests that this could be a useful step worth considering for membrane β-barrel proteins that remain challenging to work with (Carpenter *et al.*, 2008 ▸). Reproducible crystals that diffract to 2.4 Å resolution were grown for a construct comprising residues 106–430 of FapF, which represents a major step towards providing the first structural insight into the Fap machinery.

## 2. Materials and methods   

### 2.1. Macromolecular production   

Full-length FapF from *Pseudomonas* strain PAO1 was expressed as insoluble inclusion bodies and refolded from urea *via* dialysis into 20 m*M* Tris–HCl pH 8, 200 m*M* NaCl, 1% LDAO (Sigma). Based on the limited proteolysis of full-length FapF (Fig. 1 ▸), an N-terminally truncated FapF (residues 106–430; FapF^106–430^) from *Pseudomonas* strain UK4 was cloned into a pRSF-1b vector with an OmpA leader signal sequence and an N-terminal His tag.

Constructs were transformed into either *E. coli* BL21(DE3) or Lemo21 cells (New England Biolabs) and grown to an OD_600_ of 0.6–0.8 at 37°C in autoinducing TB medium before overnight induction at 25°C. Cells were harvested and resuspended in 20 m*M* Tris–HCl pH 8, 1 µg ml^−1^ DNaseI and PMSF, followed by lysis by cell disruption (Constant Systems) at 172 MPa pressure and centrifugation at 14 000 rev min^−1^ for 20 min (45 Ti rotor, Beckman). The outer membrane fraction was prepared by centrifugation of the supernatant at 100 000*g* for 2 h (45 Ti rotor; Beckman) followed by resuspension of the pellet in 20 m*M* Tris–HCl pH 8, 0.5% sarcosine (Thermo Fisher), stirring at room temperature for 30 min, a second spin at 100 000*g* for 1.5–2 h and resuspension of the pellet for overnight extraction at 4°C with 20 m*M* Tris–HCl pH 8, 200 m*M* NaCl, 1% *N*,*N*-dimethyldodecylamine *N*-oxide (LDAO; Sigma). FapF^106–430^ was then purified from the outer membrane fraction by nickel-affinity chromatography. The column was washed with 30 ml 20 m*M* Tris–HCl pH 8.0, 200 m*M* NaCl, 0.1% LDAO, 0.5% C8E4 (Generon), 20 m*M* imidazole, washed again with 30 ml 20 m*M* Tris–HCl pH 8.0, 200 m*M* NaCl, 0.5% C8E4 (Generon), 20 m*M* imidazole and eluted with 20 m*M* Tris–HCl pH 8.0, 200 m*M* NaCl, 0.5% C8E4 (Generon), 500 m*M* imidazole. The eluted fractions were gel-filtrated into 20 m*M* Tris–HCl pH 8.0, 150 m*M* NaCl, 0.5% C8E4 using a Superdex 200 column (GE Healthcare) and the fractions corresponding to the main elution peak at ∼65 ml were collected and concentrated to 10 mg ml^−1^. See Table 1 ▸ for macromolecule-production information.

### 2.2. Crystallization   

Conditions for crystallization of FapF^106–430^ were initially screened by the sitting-drop vapour-diffusion method at 20°C using sparse-matrix crystallization kits from Hampton Research and Molecular Dimensions in MRC 96-well optimization plates (Molecular Dimensions), with 100 nl protein solution and 100 nl reservoir solution, using a Mosquito nanolitre high-throughput robot (TTP Labtech). Protein crystals were obtained in a reproducible manner from 100 m*M* sodium citrate, 20–30%(*w*/*v*) PEG 400, 100 m*M* NaCl. These were manually optimized, screening over sodium citrate in the pH range 5.5–6.5 in one dimension and an NaCl concentration gradient of 50–100 m*M* in the second dimension using MRC 96-well plates with up to 400 nl protein solution and 400 nl reservoir solution. See Table 2 ▸ for crystallization information.

### 2.3. Data collection and processing   

Crystals were mounted in a MicroLoop (MiTeGen) and immediately flash-cooled in liquid nitrogen. Diffraction data from a single native crystal were collected on beamline I03 of the Diamond Light Source (DLS), England. The data were processed with *XDS* (Kabsch, 2010 ▸) and scaled using *SCALA* (Evans, 2006 ▸) within the *xia*2 package (Winter *et al.*, 2013 ▸). Data-collection statistics are shown in Table 3 ▸. The content of the unit cell was analyzed using the Matthews coefficient (Matthews, 1968 ▸). Molecular-replacement (MR) attempts were carried out using complete structures and polyalanine models with and without loop truncations of available β-barrel crystal structures in the Protein Data Bank (http://www.rscb.org) as well as using idealized models of 8–16-stranded β-barrels using C^α^ traces of various tilt angles generated using in-house scripts. MR attempts were performed using *MOLREP* (Vagin & Teplyakov, 2010 ▸) and *Phaser* (McCoy *et al.*, 2007 ▸). High-resolution data were used between 2.5 and 6 Å.

## 3. Results and discussion   

**Figure 2:**
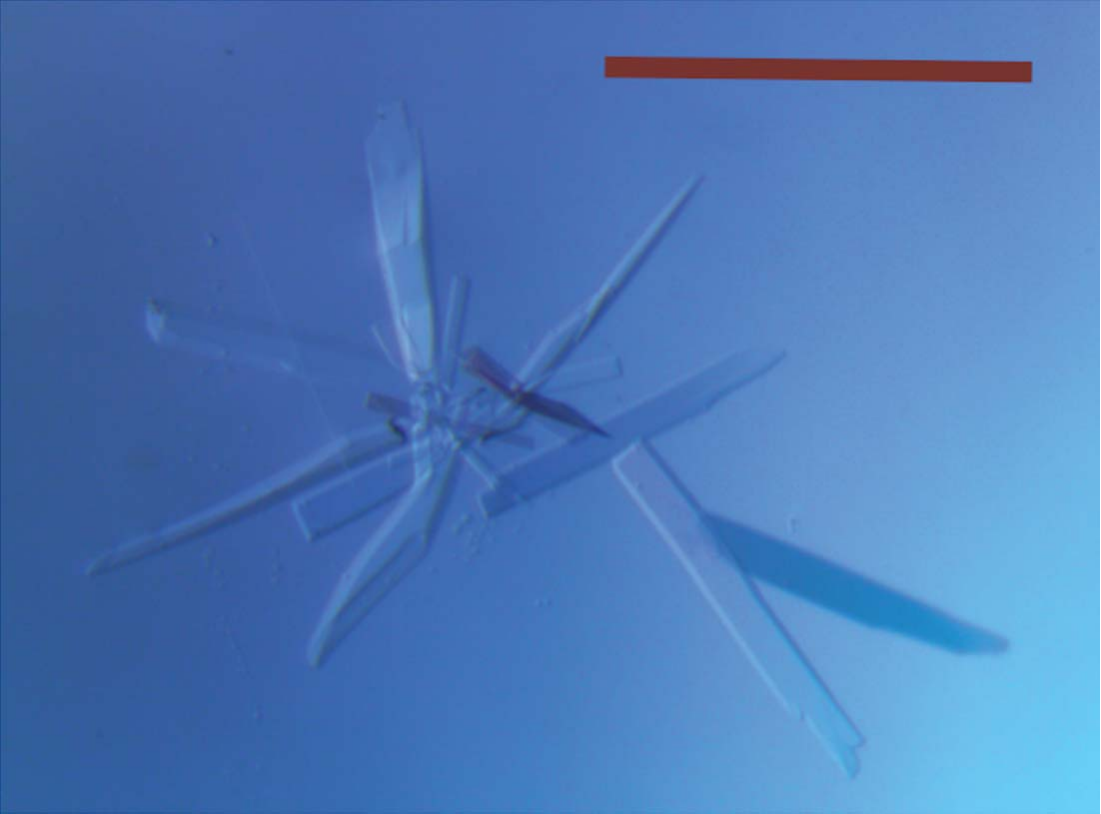
Representative native crystals of FapF^106–430^. Crystals formed over 5–7 d in the reservoir condition 100 m*M* sodium citrate, 20–30%(*w*/*v*) PEG 400, 100 m*M* NaCl. Purified FapF^106–430^ in 20 m*M* Tris–HCl pH 8.0, 150 m*M* NaCl, 0.5% C8E4 was concentrated to 10 mg ml^−1^. Scale bar 500 µm.

To date, no structural information is available for any of the components of the *fap* operon. Refolding attempts using full-length and N-terminally truncated FapF yielded microcrystals that could not be further optimized for crystallography despite exhaustive attempts. We therefore isolated and purified the FapF^106–430^ construct from the physiologically relevant environ­ment of the outer membrane (Fig. 1 ▸) and subsequently obtained suitably sized crystals (Fig. 2 ▸).

After the screening of various detergents for both extraction efficiency and appearance in gel-filtration profiles, we found that the crucial step in obtaining reproducible crystals was to reduce the concentration of LDAO in buffers to zero during nickel purification and gradually replace it with C8E4. Mixtures of LDAO and C8E4 have proven to be successful in several recent membrane-protein crystallography studies (see, for example, Goyal *et al.*, 2014 ▸; van den Berg *et al.*, 2015 ▸). Under these purification conditions crystals grew readily to up to 200 µm^3^ over the course of 5–7 d.

**Figure 3:**
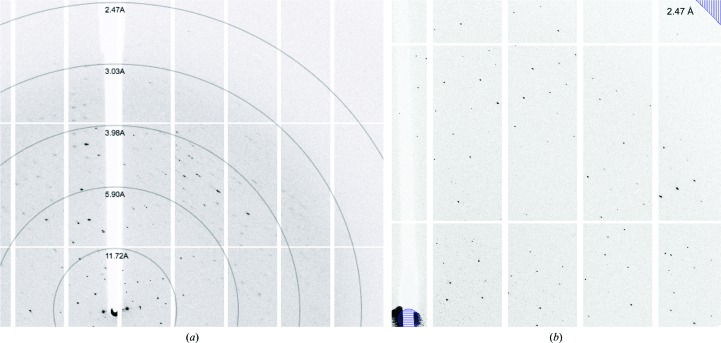
Diffraction images from a FapF^106–430^ crystal. (*a*) Representative diffraction image indicating resolution rings. (*b*) Enlarged image indicating the final data-processing resolution limit at 2.5 Å. Images were generated in *iMosflm* (Battye et al., 2011 ▸).

Diffraction data were collected to 2.36 Å resolution (Fig. 3 ▸) and indexed in space group *C*121. Data were finally scaled at 2.5 Å resolution. Analysis of the crystal content indicated that there are three to five molecules in the asymmetric unit, with a Matthews coefficient in the range 3.5–2.1 Å^3^ Da^−1^ and a corresponding solvent content in the range 65–41%; however, self-rotation analysis indicated threefold symmetry. This, combined with the fact that membrane-protein crystals tend to have a higher solvent content than their soluble counterparts owing to the presence of detergent species, leads us to believe that it is almost certainly a trimer that is present with 65% solvent content. *SFCHECK* (Vaguine *et al.*, 1999 ▸) suggests that twinning is not present (〈*E* ^4^〉/〈*I* ^2^〉 = 2.35). Data-collection and processing statistics are listed in Table 3 ▸.

Molecular-replacement attempts were made using idealized model barrels and 12-stranded and 14-stranded β-barrel structures as search models: TodX (PDB entry 3bs0; Hearn *et al.*, 2008 ▸), FadL (PDB entry 1t16; van den Berg *et al.*, 2004 ▸), NanC (PDB entry 2wir; Wirth *et al.*, 2009 ▸), TolC (PDB entry 1ek9; Koronakis *et al.*, 2000 ▸), NalP (PDB entry 1uyn; Oomen *et al.*, 2004 ▸) and COG4313 (PDB entry 4rl8; van den Berg *et al.*, 2015 ▸). No solutions were found, which was not unexpected as the sequence identities between FapF and all template models available were below 20%. We are currently preparing selenomethionine-substituted and heavy-atom derivatives in order to obtain accurate phases using anomalous dispersion techniques.

Our work involving the extensive optimization of extraction and purification protocols demonstrated that a reduction in LDAO during the early nickel-affinity purification steps was essential to obtain well diffracting crystals. It appears that gel filtration into C8E4 alone is not sufficient to remove the comparatively ‘sticky’ detergent LDAO (Newstead *et al.*, 2008 ▸). Presumably, the zwitterionic nature of LDAO is more disruptive to protein packing interactions within crystals than the milder, uncharged C8E4 (le Maire *et al.*, 2000 ▸). LDAO and C8E4 make up the majority of detergents used for outer membrane protein crystal structures. We suggest that extraction by LDAO, followed by buffer exchange into C8E4 as the sole detergent for downstream crystallization, is a worthwhile combination together with limited proteolysis when considering β-barrel membrane-protein preparations for crystal trials.

## Figures and Tables

**Table 1:** Macromolecule-production information

	Full-length FapF	Truncated FapF
Source organism	*Pseudomonas* PAO1	*Pseudomonas* UK4
DNA source	*Pseudomonas* PAO1 gDNA	pMMB190Ap:UK4fapA-F
Forward primer	TACTTCCAATCCATGGCGACGGAATCCGAG	GGCCGGTACCAAGGATGATTCGGAGCCGGC
Reverse primer	TATCCACCTTTACTGTCAGAAGTAGTAGGGGAATTT	CCGGAAGCTTTTAGAAGTAGTACGGGAATTTCAGGC
Cloning vector	pNIC-NTH	pRSF-1b
Expression vector	pNIC-NTH	pRSF-1b
Expression host	*E. coli* BL21(DE3)	*E. coli* Lemo21
Complete amino-acid sequence of the construct produced	MHHHHHHHSSGVDLGTENLYFQSMATESEVEALKKELLELRQRYEAQQNALMVLEQRVRQVEAQPQAPQPQRLVKSIQPPAQARNDANAVAGTYGASLKDDGAPAPSVENIYQDASGFFGGGTFSLETGLTYSHYDTRQLFLNGFLALDSIFLGNIGVDQIDADIWTLDLTGRYNWNQRWQVDINAPVVYRESTYQSAGAGGSTSQITEKSVTGDPRLGDVSFGVAYKFLDESESTPDAVVSLRVKAPTGKDPYGIKLKQVPGNNNLNVPDDLPTGNGVWSITPGISLVKTVDPAVLFGSLSYTYNFEESFDDINPQQGVKTGGKVKLGNWFQLGVGVAFALNEKMSMSFSFSELISQKSKVKQDGQSWQTVSGSDANAGYFGLGMTYAVSNRFSIVPSLSIGITPDAPDFTFGVKFPYYF	MKKTAIAIAVALAGFATVAQATSHHHHHHGTKDDSEPAQSVSNLYNEASGFFGNGKFSFETGITYARYDARQLTLNGFLALDSIFLGNINLDRIKADNWTLDLTGRYNLDNRWQFDVNVPVVYRESTYQSGGASGGDPQATSEESVSRDPTIGDVNFGIAYKFLDESATMPDAVVSVRVKAPTGKEPFGIKLVRSTANDNLYVPESLPTGNGVWSITPGLSLVKTFDPAVLFGSVSYTHNLEDSFDDISSDVNQKVGGKVRLGDSFQFGVGVAFALNERMSMSFSVSDLIQRKSKLKPDGGGWQSIVSSDANAGYFNVGMTIAASENLTIVPNLAIGMTDDAPDFTFSLKFPYYF

**Table 2:** Crystallization

Method	Sitting-drop vapour diffusion
Plate type	24-well cell-culture plate
Temperature (K)	293
Protein concentration (mg ml^−1^)	10
Buffer composition of protein solution	20 m*M* Tris–HCl pH 8, 150 m*M* NaCl, 0.5% C8E4
Composition of reservoir solution	100 m*M* sodium citrate, 20–30%(*w*/*v*) PEG 400, 100 m*M* NaCl
Volume and ratio of drop	800 nl, 1:1
Volume of reservoir (µl)	80

**Table 3:** Data collection and processing Values in parentheses are for the outer shell.

Diffraction source	I04, DLS
Wavelength (Å)	0.97980
Temperature (K)	100
Detector	PILATUS 6M
Crystal-to-detector distance (mm)	496.43 (from the image header)
Rotation range per image (°)	0.2
Total rotation range (°)	180
Exposure time per image (s)	0.1
Space group	*C*121
*a*, *b*, *c* (Å)	143.39, 124.56, 80.37
α, β, γ (°)	90.00, 96.32, 90.00
Resolution range (Å)	50.49–2.36 (2.42–2.36)
Total No. of reflections	192836 (12153)
No. of unique reflections	57290 (4212)
Completeness (%)	99.4 (95.3–99.0)
Multiplicity	3.4 (2.9)
〈*I*/σ(*I*)〉	9.4 (1.2)
*R*_merge_ †	0.077 (0.492)
Overall *B* factor from Wilson plot (Å^2^)	44.86

† *R*_merge_ = 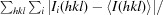 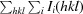, where 〈*I*(*hkl*)〉 is the mean intensity of the observations *I_i_*(*hkl*) of reflection *hkl*.
